# ADAMTS7 degrades Comp to fuel BMP2‐dependent osteogenic differentiation and ameliorate oncogenic potential in osteosarcomas

**DOI:** 10.1002/2211-5463.12939

**Published:** 2020-08-10

**Authors:** Chao Wang, Yunqing Chen, Hongfei Xiang, Xiaolin Wu, Qian Tang, Xuexiao Ma, Lu Zhang

**Affiliations:** ^1^ Department of Spine Surgery The Affiliated Hospital of Qingdao University China; ^2^ Department of Pathology The Affiliated Hospital of Qingdao University China; ^3^ Department of Medical Genetics The Affiliated Hospital of Qingdao University China; ^4^ Medicine Research Center The Affiliated Hospital of Qingdao University China

**Keywords:** ADAMTS, BMP2, Comp, osteogenic differentiation, osteosarcoma

## Abstract

Osteosarcoma (OS) is the most common primary malignant bone tumor in children and adolescents, with a high metastatic potential. Despite dramatic changes in OS treatments over the past decades, their efficiency still remains limited, with severe complications and adverse side effects. Key mechanisms underlining tumorigenesis, metastasis and chemotherapy resistance are currently lacking, in turn hindering any progress with respect to developing effective and safe therapeutic strategies against OS. Recently, ADAMTS7, a member of the disintegrin and metalloproteinase with thrombospondin motifs (ADAMTS) family, was shown to be involved in osteogenic differentiation‐related pathological processes. ADAMTS7 promotes vascular calcification via disturbing the balance between osteogenic bone morphogenetic protein (BMP)2 (regulating osteogenic differentiation and bone formation during development) and its natural inhibitor cartilage oligomeric matrix protein (Comp). Hence, in the present study, we aimed to investigate the role of ADAMTS7 in the pathological process of OS. We first revealed that ADAMTS7 was decreased in OS tissues. Lower expression of ADAMTS7 was correlated with poor histological differentiation and an advanced clinical stage of OS. Through loss‐ and gain‐function analysis, we further revealed that ADAMTS7 attenuated cell proliferation, migration and invasion, at the same time as promoting the expression of osteogenic differentiation markers in two OS cell lines: MG63 and SAOS2. Moreover, Comp was responsible for the effects of ADAMTS7 on OS pathogenesis by reinforcing cell osteogenic differentiation mediated by BMP2 *in vitro*. In conclusion, ADAMTS7‐mediated degradation of Comp may provide a potential therapeutic target for the treatment of OS.

## Graphical abstract

Loss of differentiation and lack of osteoblast marker expression are prominent features of osteosarcoma (OS). Here, we report that the expression of metalloproteinase ADAMTS7 is decreased in OS tissues and associated with adverse OS clinical outcomes. Moreover, we showed ADAMTS7 can bind and degrade cartilage oligomeric matrix protein to induce osteogenic differentiation and inhibit an OS malignant phenotype in a bone morphogenetic protein 2‐dependent manner.

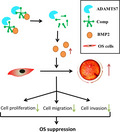

**Abbreviations**
ANOVA: analysis of variance
ADAMTS: a disintegrin and metalloproteinase with thrombospondin motifs
BMP: bone morphogenetic protein
CCK‐8: Cell Counting Kit‐8
Comp: cartilage oligomeric matrix protein
FACS: fluorescence activated cell sorting
IHC: immunohistochemistry
NC: adjacent tissues
OS: osteosarcoma
siRNA: small interfering RNA

Osteosarcoma (OS) is the most common primary malignant bone tumor in children and adolescents [1]. It has a high metastatic potential and approximately 20% of patients develop lung metastases [2, 3]. The pivotal mechanisms of tumorigenesis, local invasion, metastasis and chemotherapy resistance remain largely unknown [4, 5]. Despite the treatments for OS being updated and having undergone dramatic changes over recent decades, the efficiency of current therapeutic strategies is still limited, accompanied by severe complications and adverse effects [6, 7]. Therefore, effective and safe therapeutic strategies on OS are urgently needed.

Loss of differentiation and absent expression of osteoblast markers are prominent features of OS [8]. More than 80% of OS cases are graded histopathologically as poor‐differentiated OS, and the well‐differentiated tumors are usually classified as the low‐grade subtype [9, 10]. Previous studies have reported that the interruption of osteogenic differentiation could result in the progression of OS, whereas promotion of osteoblast differentiation could arrest tumor growth and metastasis, as a potential therapeutic strategy for OS [11, 12]. Bone morphogenetic proteins (BMPs) play a crucial role in regulating osteogenic differentiation and bone formation during development, healing, and bone turnover [13]. It has been reported that BMP2 can induce bone formation and inhibit tumorigenicity of cancer stem cells in human OS cell line [14]. Furthermore, genetic mutations of the gene for BMP2 were associated with a higher OS risk and poorer prognosis in a Chinese population [15].

Recently, ADAMTS7, a member of a disintegrin and metalloproteinase with thrombospondin motifs (ADAMTS) family, was confirmed to be involved in osteogenic differentiation‐related pathologic processes [16]. ADAMTS7 directly binds with and degrades cartilage oligomeric matrix protein (Comp), a prominent non‐collagenous component of cartilage, accelerating the progression of both surgically induced and collagen‐induced arthritis [17, 18]. ADAMTS7 is also a direct target of parathyroid hormone‐related peptide and negatively intercedes chondrocyte differentiation by interaction with granulin/epithelin precursor [19]. In addition, ADAMTS7 and ADAMTS12 appear to be potent negative regulators of endplate cartilage development. *Adamts7^−/−^ Adamts12^−/−^* hindlimbs develop heterotopic tendon and ligament ossification [20]. Moreover, ADAMTS7 promotes vascular calcification by disturbing the balance between osteogenic BMP2 and its natural inhibitor Comp [21]. However, whether ADAMTS7 affects the pathology of OS and the underlying mechanism remains unknown. In the present study, we report that ADAMTS7 can bind and degrade Comp to induce osteogenic differentiation and inhibit OS malignant behaviors in a BMP2‐dependent manner.

## Materials and methods

### Patients and samples

Samples of both OS tissues and adjacent tissues were obtained from 49 patients diagnosed as OS from the Affiliated Hospital of Qingdao University between January 2013 and December 2017, with written informed consent having been obtained from the patients or their guardians. All of the patients underwent surgical resection and the tissue samples were immediately preserved in liquid nitrogen until further use. The research was approved by Medical Ethics Committee of Qingdao University. All subjects provided their written informed consent in accordance with the Declaration of Helsinki.

### RNA extraction and RT‐PCR

Total RNA was extracted from tissues or cultured cells by TRIzol reagent (Invitrogen, Thermo Fisher Scientific, Inc., Waltham, MA, USA), after which cDNA was synthesized from 1 μg total RNA using a Reverse Transcription kit (Takara Biotechnology, Shiga, Japan) in accordance with the manufacturer’s instructions. A quantitative PCR was performed with a LightCycler 480 Realtime PCR System (Roche, Mannheim, Germany) using the following protocol: 95 °C for 3 min, followed by 40 cycles of 95 °C for 15 s and 60 °C for 30 s. The results were normalized to the expression of *β‐actin*. The primers used were: *ADAMTS7* (forward primer, 5′‐GTGGAGACCCTGGTAGTAGC‐3′ and reverse primer, 5′‐TCTGCGTGGTGCGTGATCTTTA‐3′); *BMP2* (forward primer, 5′‐TGACGAGGGTCCTGAGCGA‐3′ and reverse primer, 5′‐CCTGAGTGCCTGCGATACA‐3′); *Runx2* (forward primer, 5′‐CTGGTGTCTCGGCTTCAATCT‐3′ and reverse primer, 5′‐TTCAAGGTGCCAAGAGGTAAGT‐3′); *Msx2* (forward primer, 5′‐CCGCCTGTGGGACTCTATG‐3′ and reverse primer, 5′‐GGCTGGTACTGCCTTCGTG‐3′); *Osteocalcin* (*OCN*) (forward primer, 5′‐AGGGCAGCGAGGTAGTGAA‐3′ and reverse primer, 5′‐TCCTGAAAGCCGATGTGGT‐3′); *Osteopontin* (*OPN*) (forward primer, 5′‐AGCAGCAGGAGGAGGCAGAGCACAG‐3′ and reverse primer, 5′‐CTGTGCTCTGCCTCCTCCTGCTGCT‐3′); *β‐actin* (forward primer, 5′‐ATCTGGCACCACACCTTC‐3′ and reverse primer, 5′‐AGCCAGGTCCAGACGCA‐3′).

### Western blotting

Proteins mixed with sample buffer were resolved by 10% or 12% SDS/PAGE after boiling at 95 °C for 10 min. Subsequently, proteins were transferred onto poly(vinylidene difluoride) membranes. The membranes were blocked by 5% skim milk for 60 min. Anti‐ADAMTS7 (#DF9177, dilution 1:1000; Affinity Bioreagents, Golden, CO, USA), anti‐Comp (#ab74524, 1:1000; Abcam, Cambridge, MA, USA), anti‐BMP2 (#sc‐6895, dilution 1:200; Santa Cruz Biotechnology, Santa Cruz, CA, USA), anti‐Runx2 (#12556S, dilution 1:1000; Cell Signaling Technology, Beverly, MA, USA) and anti‐β‐actin (#3700S, dilution 1:2000; Cell Signaling Technology) were incubated with the membranes overnight at 4 °C Then, horseradish peroxidase conjugated anti‐rabbit or anti‐goat IgG were used as secondary antibodies and the immunoblots were visualized using ECL Reagents (#WBKLS0100; Millipore, Burlington, MA, USA).

### Immunohistochemistry

Paraffin‐embedded sections of OS tissues were deparaffinized and rehydrated. Slides were boiled in citrate buffer at 95 °C for 15 min to retrieve antigen. The sections were incubated in 3 % H_2_O_2_ in methanol for 10 min and blocked with normal goat serum at 37 °C for 1 h, then incubated with primary ADAMTS7 antibody (#ab203027, dilution 1:100; Abcam) at 4 °C overnight. After incubated with goat anti‐rabbit IgG (Zhongshan Golden Bridge Co., Beijing, China) for 1 h at room temperature, the sections were stained with 2,4‐diaminobutyric acid working reagent (Zhongshan Golden Bridge Co.). All images were captured using a microscope (Olympus, Tokyo, Japan) and positive staining intensity was measured by imagej (NIH, Bethesda, MD, USA).

### Cell culture and transfection

OS cells line MG63 and SAOS2 were obtained from Cell Bank, Chinese Academy of Sciences (Beijing, China). Cells were maintained and cultured according to the manufacturer’s protocol with 10% FBS (Biological Industries, Beit HaEmek, Israel) and incubated at 37 °C in a 5% CO_2_ humidified incubator. Recombinant human Comp (rComp; #10173‐H08H) was purchased from Sino Biological (Shanghai, China) and Noggin (#120‐10C) was obtained from PeproTech (Rehovot, Israel).

ADAMTS7‐small interfering RNA (siRNA) and negative controls were synthesized by GenePharma (Shanghai, China). The sequence of ADAMTS7‐siRNA was 5′‐ACCUAAAGAUCACGCACCATT‐3′ and the nonsilencing siRNA (Scramble‐siRNA) oligonucleotide was used as a negative control. The cDNA encoding human full‐length ADAMTS7 was amplified by PCR and subcloned into the pcDNA3.1 vector to construct the pcDNA_ADAMTS7_ vector. The empty pcDNA3.1 vector was used as a negative control. All the transfections were performed using Lipofectamine 3000 (Invitrogen, Carlsbad, CA, USA) in accordance with the manufacturer’s instructions.

### Cell proliferation assay

MG63 or SAOS2 cells were trypsinized to single‐cell suspension and transferred to a 96‐well plate (3 × 10^3^ cells per well). Cells were then treated with serum‐free Dulbecco's modified Eagle's medium for synchronization. In different time points, Cell Counting Kit‐8 (CCK‐8) reagent was added and incubated for 4 h. *D*_450_ was read using a microplate reader (Varioskan Flash; Thermo Fisher Scientific, Inc.).

Osteosarcoma cells were trypsinized and fixed with 70% ethanol, followed by treatment with propidium iodide (20 μg·mL^−1^) and RNase A (500 mg·mL^−1^). Then, fluorescence‐activated cell sorting (FACS) was performed to analyze the cell cycle. Each experiment was performed three times independently.

### Scratch wound healing assay

MG63 or SAOS2 were seeded in 24‐well plates and transfected. The medium was changed to serum‐free Dulbecco's modified Eagle's medium overnight for synchronization. Then, scratching was performed to make a gap and fresh medium that contained 2% FBS was added. Four fields were randomly selected in each well to record gap distances via a light microscope at different time points.

### Transwell assay

Cell migration was assessed in 8‐μm transwell chambers (Costar Inc., Cambridge, MA, USA) in accordance with the manufacturer's instructions. OS cells were seeded at 2.5 × 10^4^ cells per well followed by incubation for 16 h at 37 °C. For the cell invasion assay, 1 × 10^5^ cells were added to Matrigel (BD Biosciences, Franklin Lakes, NJ, USA) coated chambers for 48 h in accordance with the manufacturer's instructions. After incubation, cells on the upper side were scraped off by cotton swabs, and the migrated or invasive cells on the bottom of the membrane were fixed with 4% formaldehyde for 30 min. Then, cells were stained with 0.1% crystal violet staining solution. Cells were counted in four randomly selected microscopic fields and the average numbers were calculated.

### Alizarin red staining

MG63 cells were stimulated by 0.5 mm β‐glycerophosphate (#sc‐220452; Santa Cruz Biotechnology) and 50 mg·L^−1^ l‐ascorbic acid (#A92902; Sigma‐Aldrich, St Louis, MO, USA) for 21 days to induce osteogenic differentiation. Cells were rinsed with PBS for three times, fixed with formalin for 30 min, and stained with alizarin red S (#A5533; Sigma‐Aldrich) for 15 min and washed with 0.2% acetic acid. The calcium content was analyzed using a QuantiChrom Calcium Assay Kit (BioAssay Systems, Hayward, CA, USA) and normalized by protein concentration.

### Co‐immunoprecipitation

MG63 cell were pretreated with recombinant Comp and BMP2 protein for 24 h. Then cell lysates were incubated with anti‐Comp antibody or anti‐BMP2 prior to immunoprecipitation with protein G/A agarose beads (#sc‐2003; Santa Cruz Biotechnology). The precipitated proteins were resolved by 12% or 10% SDS/PAGE and immunoblotted with anti‐BMP2 antibody or anti‐Comp antibody, respectively. Rabbit or goat IgG antibodies served as a negative control.

### Statistical analysis

All results were expressed as the mean ± SEM. Statistical analysis included the use of paired *t*‐test for comparison of ADAMTS7 and Comp expression in OS and noncancerous tissues, and an unpaired Student's *t*‐test for comparison of the effects of ADAMTS7 on cell proliferation, migration and protein expression in two groups. Comparisons among more than two groups involved one‐way analysis of variance (ANOVA) followed by the Student–Newman–Keuls test for post‐hoc comparison or two‐way ANOVA with Bonferroni's test for multiple comparisons. Statistical analyses were conducted using prism, version 5.0 (GraphPad Software Inc, La Jolla, CA, USA). *P *< 0.05 was considered statistically significant.

## Results

### ADAMTS7 was down‐regulated in OS tissues

**Fig. 1:**
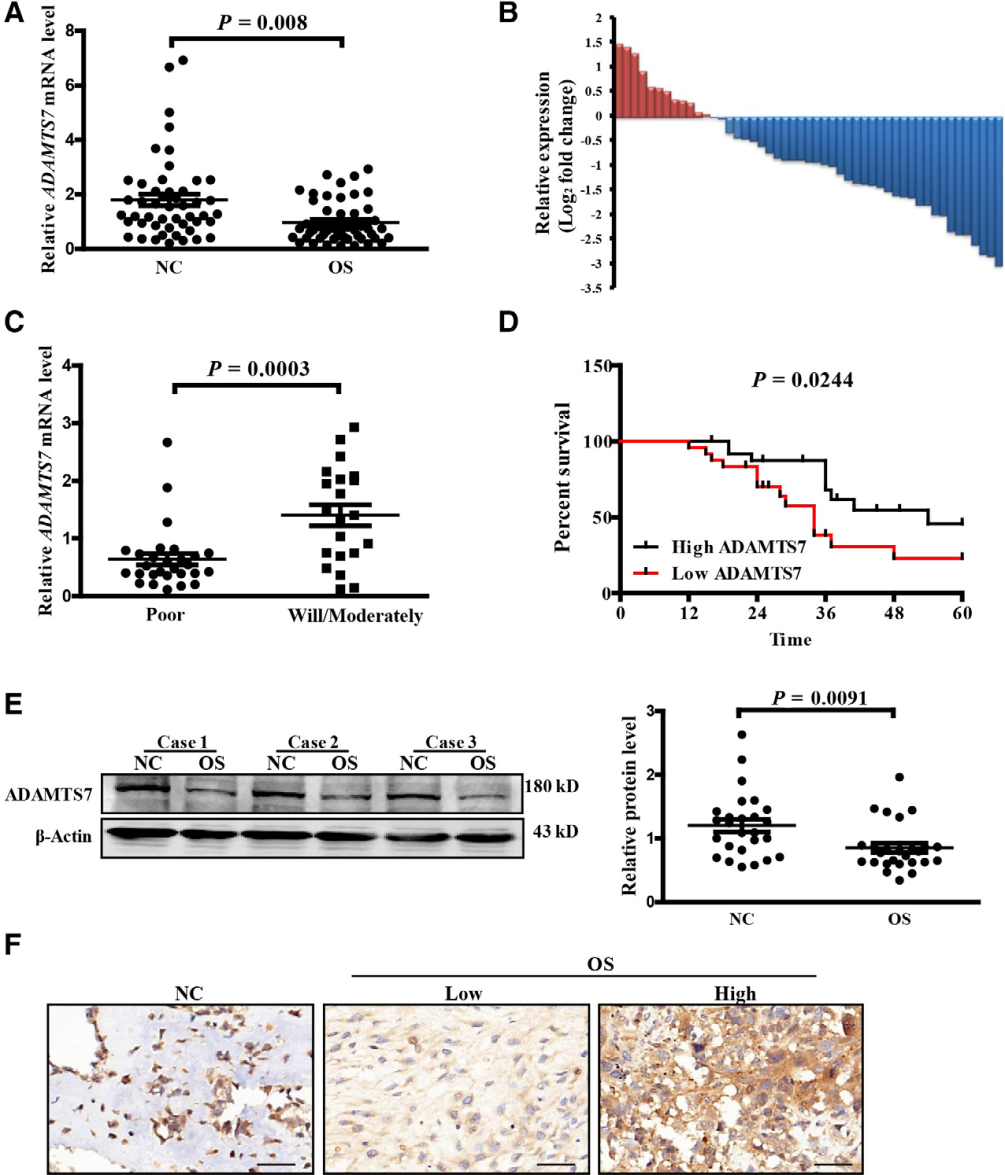
ADAMTS7 was down‐regulated in OS tissues. (A) The relative mRNA expression of *ADAMTS7* in OS compared to the corresponding adjacent tissues (*n* = 49). (B) The results are presented as the log_2_(fold‐change) in OS samples relative to the adjacent tissues. (C) The relative mRNA expression of *ADAMTS7* in poor‐ (*n* = 28) and well/moderately differentiated (*n* = 21) OS tissues. (D) Survival curve for high‐ and low‐*ADAMTS7*‐expression OS patients. *P* value by log‐rank (Mantel–Cox) test. (E) Representative images of western blotting in OS and adjacent tissues (*n* = 26). The relative expression level of ADAMTS7 was quantified. (F) Representative images of IHC in OS and adjacent tissues (200×). Scale bar = 20 μm. Data in (A), (B), (C) and (E) were analyzed by Student's *t*‐test.

To confirm whether ADAMTS7 was involved in the progression of OS, we first determined the expression of ADAMTS7 in OS tissues. Forty‐nine pairs of OS and adjacent tissues were collected and analyzed by RT‐PCR. The results showed that the mRNA expression of *ADAMTS7* significantly decreased in OS tissues compared to the adjacent tissues (NC vs. OS: 1.792 ± 0.2131 vs. 0.9677 ± 0.1110, *n* = 49, *P *= 0.0008) (Fig. 1A). As shown in Fig. 1B, ADAMTS7 was down‐regulated in most OS tissue samples (37/49; 75.51%). Based on the data obtained by RT‐PCR, the mean level of *ADAMTS7* in OS tissues was used as a cut‐off to divide cases into low or high expression group. The associations between clinicopathologic factors and *ADAMTS7* expression are listed in Table 1. Our results showed that a low *ADAMTS7* level was correlated with a poor histological differentiation level (*P *< 0.001) and advanced clinical stage (Enneking Stage System, *P *= 0.028), whereas no association was found with other clinical factors, including age, gender, tumor size and tumor location. We further divided the OS patients to two groups according to the differentiation levels and assessed *ADAMTS7* mRNA expression. As expected, patients with poor‐differentiated level also showed a significant lower level of *ADAMTS7* compared to the histologically well‐differentiated ones (Poor vs. Will/Moderately: 0.6410 ± 0.1014, *n* = 28 vs. 1.403 ± 0.1842, *n* = 21, *P *= 0.0003) (Fig. 1C). Meanwhile, we found that lower *ADAMTS7* was closely correlated with a shorter survival rate (*P *= 0.0244) (Fig. 1D). Furthermore, western blotting and immunohistochemistry (IHC) further confirmed the reduced expression of ADAMTS7 protein in OS tissues (NC vs. OS: 1.203 ± 0.099 vs. 0.8517 ± 0.0736, *n* = 26, *P *= 0.0008) (Fig. 1E,F).

**Table 1:** Correlations between clinicopathological features and the expression of ADAMTS7 in OS tissues.

Characteristics	Number of patients	Low expression	High expression	*P* value
Age (years)				
≤ 40	21	7	14	0.058
> 40	28	17	11	
Gender				
Male	28	13	15	0.680
Female	21	11	10	
Histological differentiation				
Poor	28	20	8	**< 0.001**
Well/moderately	21	4	17	
Histological subtypes				
Osteoblastic	31	14	17	0.432
Chondroblastic	8	6	2	
Fibrohistiocytic	7	3	4	
Others	3	1	2	
Tumor size (cm)				
< 8	26	14	12	0.219
≥ 8	23	10	13	
Tumor location				
Tibia/femur	27	11	16	0.498
Elsewhere	22	13	9	
Tumor stage				
IIA	18	6	12	**0.028**
IIB/III	31	18	13	

The use of bold indicates a significance difference.

### Silence of endogenous ADAMTS7 promoted growth and metastasis of OS cells

**Fig. 2:**
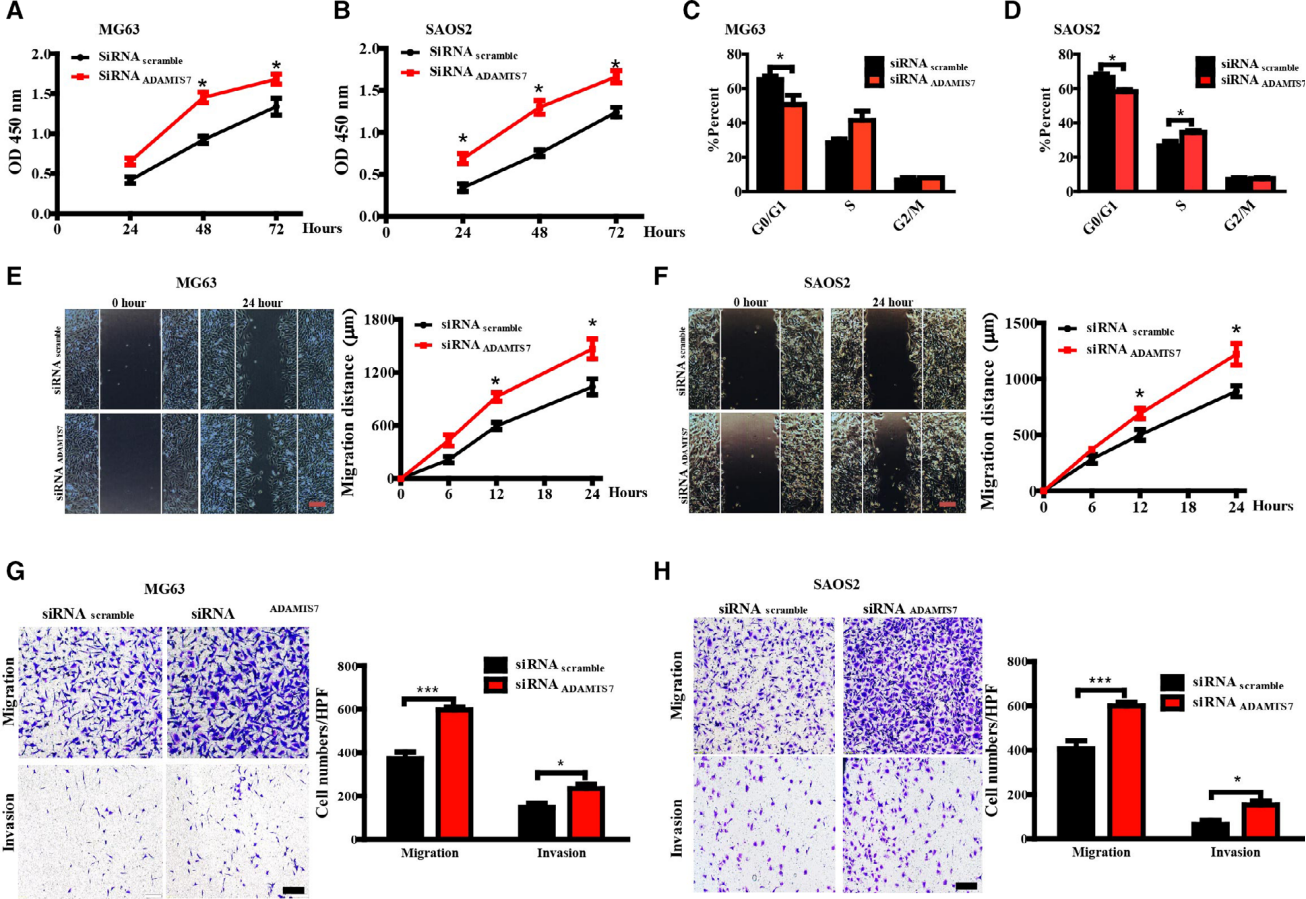
Silence of ADAMTS7 promoted the cancerous ability of OS cells. (A, B) Proliferation of OS cell lines MG63 and SAOS2 at 24, 48 and 72 h was determined by CCK‐8 after transfection with siRNA. Data are expressed as the mean ± SEM from three independent experiments performed in duplicate. *P < 0.05. (C, D) Cell‐cycle distribution AT 24 h was confirmed by propidium iodide staining and FACS analysis in MG63 and SAOS2 cells. Data are expressed as the mean ± SEM from four independent experiments. *P < 0.05. (E, F) Representative images of cell migration 24 h after scratching (magnification 100×). Scale bar = 1 mm. The mean distances migrated by cells at 6, 12 and 24 h were quantified. Data are expressed as the mean ± SEM from three independent experiments. *P < 0.05. Data in (A) to (F) were analyzed by two‐way ANOVA with Bonferroni's test for multiple comparisons. (G, H) Representative images for a transwell assay in MG63 and SAOS2 cells (magnification 100×). Scale bar = 200 μm. Upper: Migration. Lower: Invasion. The numbers of transmembrane cells were calculated and quantified. Data are expressed as the mean ± SEM (*n* = 3). ***P < 0.001, *P < 0.05 by two‐tailed Student's *t*‐test.

To investigate the effect of ADAMTS7 on OS, MG63 and SAOS2 cells were transfected with siRNA_ADAMTS7_ or siRNA_Scramble_ with high transfection efficiency (data not shown). As indicated by a CCK‐8 assay, the viability of MG63 or SAOS2 cells was markedly increased after ADAMTS7 knockdown (Fig. 2A,B). Flow cytometric analysis indicated that the number of OS cells in G0/G1 phase from the knockdown group was significantly lower than that from the control group (Fig. 2C,D). Additionally, a scratch wound assay revealed a dramatic increase of mean migration distance in ADAMTS7‐silencing MG63 or SAOS2 cells (Fig. 2E,F). Moreover, a transwell assay was performed with or without Matrigel barrier and indicated a stronger migration and invasion ability of siRNA_ADAMTS7_ treated cells (Fig. 2G,H). These data support the idea that ADAMTS7 specifically targeted OS proliferation and metastasis *in vitro*.

### ADAMTS7 overexpression repressed the malignant phenotypes of OS cells

**Fig. 3:**
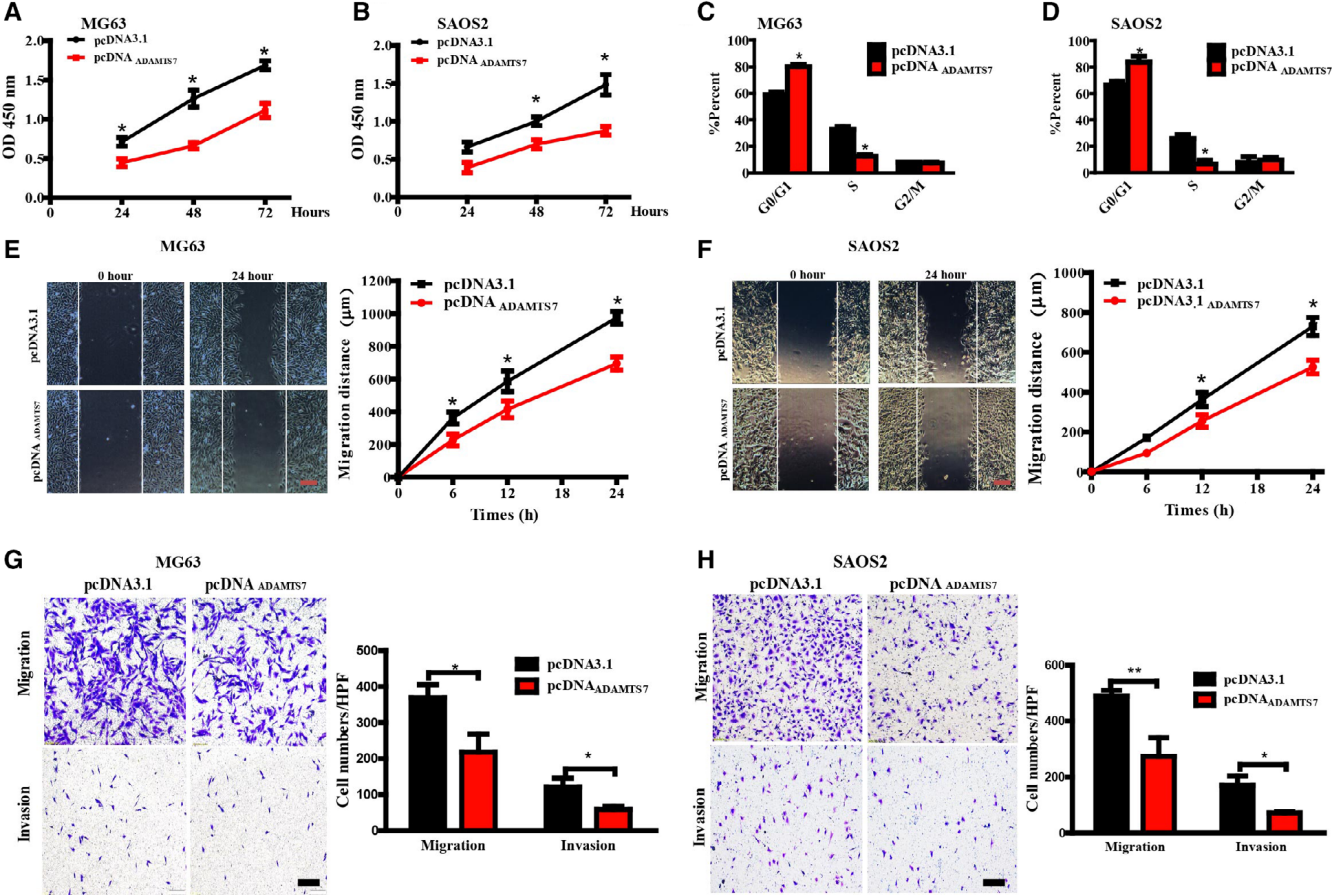
ADAMTS7 suppressed the proliferation and migration ability of OS cells. (A, B) Overexpression of ADAMTS7 attenuated the proliferation of OS cells in different time points. Data are expressed as the mean ± SEM from three independent experiments performed in duplicate. *P < 0.05. (C, D) Cell‐cycle distribution was confirmed by FACS in ADAMTS7 overexpressing MG63 and SAOS2 cells. Data are expressed as the mean ± SEM from three independent experiments. *P < 0.05. (E, F) Representative images of cell migration 24 h after scratching (magnification 100×). Scale bar = 1 mm. The mean distances migrated at different time points were quantified as the mean ± SEM (*n* = 3). *P < 0.05. Data in (A) to (F) were analyzed by two‐way ANOVA with Bonferroni's test for multiple comparisons. (G, H) Representative images of a transwell assay (magnification 100×). Scale bar = 200 μm. The numbers of transmembrane cells were quantified for thee independent experiments and expressed as the mean ± SEM. **P < 0.01, *P < 0.05 by a three‐tailed Student's *t*‐test.

Reciprocally, we investigated whether ADAMTS7 overexpression played an inhibitory role in OS progression. MG63 or SAOS2 cells were transfected with pcDNA_ADAMTS7_ plasmid and gene overexpression was confirmed by RT‐PCR and western blotting (data not shown). Cell viability was significantly suppressed in ADAMTS7 overexpressing group (Fig. 3A,B). Meanwhile, cell cycles of ADAMTS7 overexpressing cells were mostly blocked in G0/G1 phase compared to the control cells (Fig. 3C,D). Scratch wound healing and transwell assays both showed an attenuated migration and invasion capacity after ADAMTS7 overexpression: MG63 (Fig. 3E,G) and SAOS2 (Fig. 3F,H). Taken together, these data were in accordance with that found in the ADAMTS7 deficiency group and provide support for the protective effect of ADAMTS7 in OS.

### ADAMTS7 facilitated osteogenic differentiation of OS cells via a BMP2‐related pathway

**Fig. 4:**
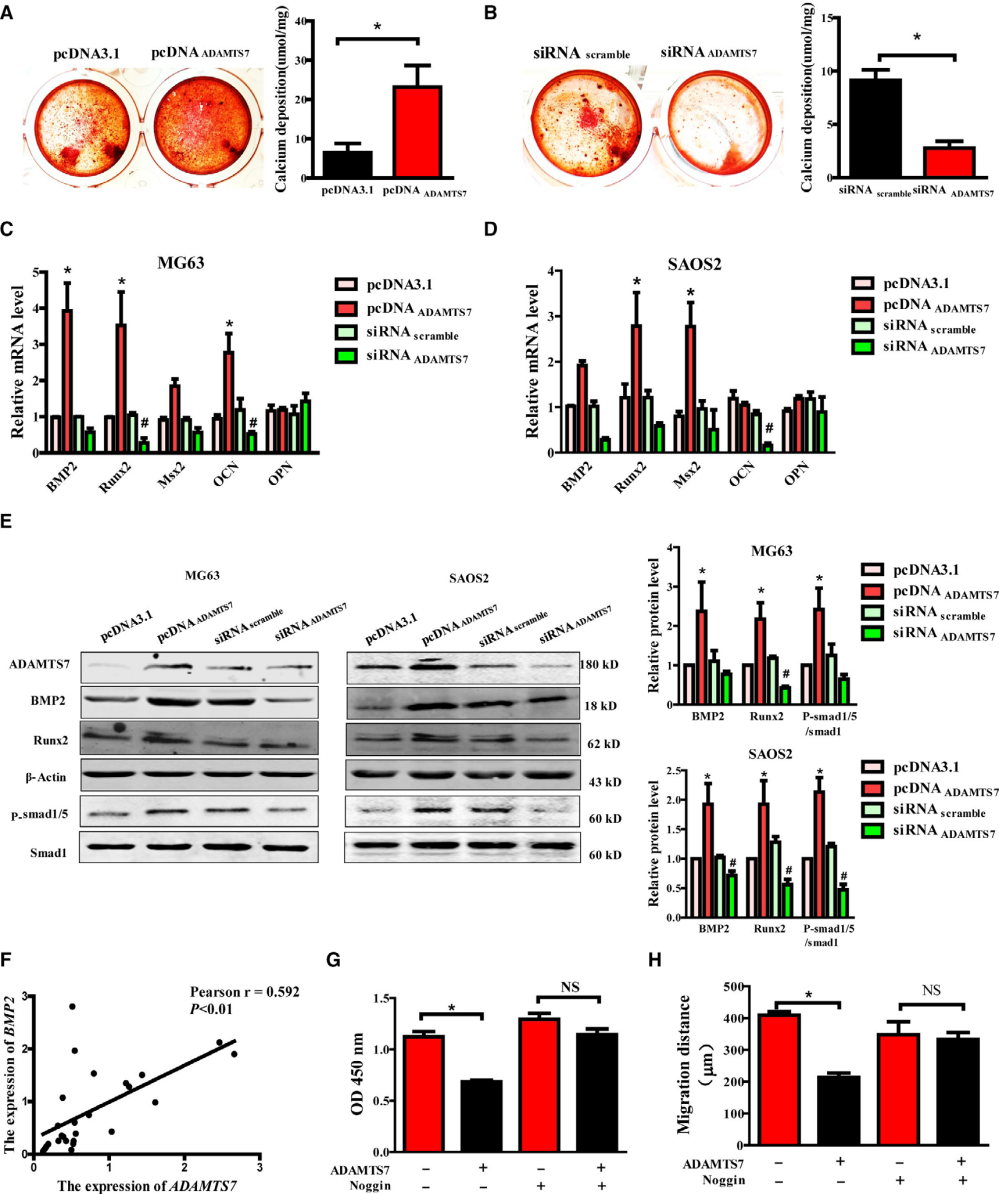
ADAMTS7 reinforced osteogenic differentiation and suppressed OS pathogenesis via BMP2. (A, B) Representative images of biomineralization by alizarin red staining in MG63 cells stimulated by ascorbic acid and β‐glycerophosphate for 21 days with ADAMTS7 overexpressing (A) or silencing (B). Calcium deposition was analyzed and quantified as the mean ± SEM (*n* = 3). *P < 0.05 two‐tailed Student's *t*‐test. (C, D) RT‐PCR analysis in different treated OS cells revealed altered mRNA expression of genes involved in osteoblast differentiation. Data are expressed as the mean ± SEM from three independent experiments performed in duplicate. *P < 0.05 compared to the pcDNA3.1 group. ^#^ *P *< 0.05 compared to the siRNA_scrmable_ group. (E) Representative images for western blotting of osteogenic protein in transfected OS cells. Left: MG63 cells. Right: SAOS2 cells. The relative expression of BMP2, Runx2 and p‐Smad1/5 was quantified as the mean ± SEM for three independent experiments. *P < 0.05 compared to the pcDNA3.1 group. ^#^ *P *< 0.05 compared to the siRNA_scrmable_ group. Data in (C) to (E) were analyzed by two‐way ANOVA with Bonferroni's test for multiple comparisons. (F) Correlation of ADAMTS7 and BMP2 protein in OS tissues. (G) Proliferation and (H) migration ability were assessed in ADAMTS7 overexpressing cells with or without Noggin (100 ng·mL^−1^) stimulation. Data are expressed as the mean ± SEM (*n* = 3). *P < 0.05. NS, not significant by one‐way ANOVA followed by the Student–Newman–Keuls test for post‐hoc comparison.

To investigate the potential mechanisms underlying the inhibitory effects of ADAMTS7 on growth and metastasis of OS cells, we analyzed whether ADAMTS7 contributed to osteogenic differentiation and osteoblast markers expression in OS. Mineralized calcium phosphate nodule formation in MG63 cultured in the presence of ascorbic acid and β‐glycerophosphate was analyzed by alizarin red staining. We found that overexpression of ADAMTS7 increased biomineralization in MG63 cells treated with osteogenic stimulation (Fig. 4A) and also that suppression of ADAMTS7 expression could inhibit biomineralization (Fig. 4B). Furthermore, the expression of osteoblast markers was analyzed in both MG63 and SAOS2 cells. The mRNA levels of osteogenic‐related molecules were uniformly increased in ADAMTS7 overexpressing cells and decreased in ADAMTS7 silencing OS cells (Fig. 4C,D). Meanwhile, the relative protein levels of BMP2 and Runx2, as well as p‐Smad1/5, were significantly higher in ADAMTS7 overexpressing cells, whereas they were lower in siRNA_ADAMTS7_ transfected cells (Fig. 4E).

Among these osteoblast markers, we found the expression of BMP2, a key osteogenic factor in many physiological and pathogenic processes, was positively correlated with the expression level of ADAMTS7 in OS tissues (Pearson *r* = 0.592, *P *< 0.01) (Fig. 1F). Then, we treated MG63 cells with Noggin, an inhibitor of BMP2, to assess the contribution of BMP2 in ADAMTS7‐mediated OS progression. As expected, preconditioning with Noggin significantly reversed the negative effects of ADAMTS7 on MG63 cell growth and metastasis (Fig. 4G,H). Taken together, these data indicate that ADAMTS7 restrained the malignant processes of OS cells via the BMP2‐related pathway.

### ADAMTS7 degraded Comp in OS cells

**Fig. 5:**
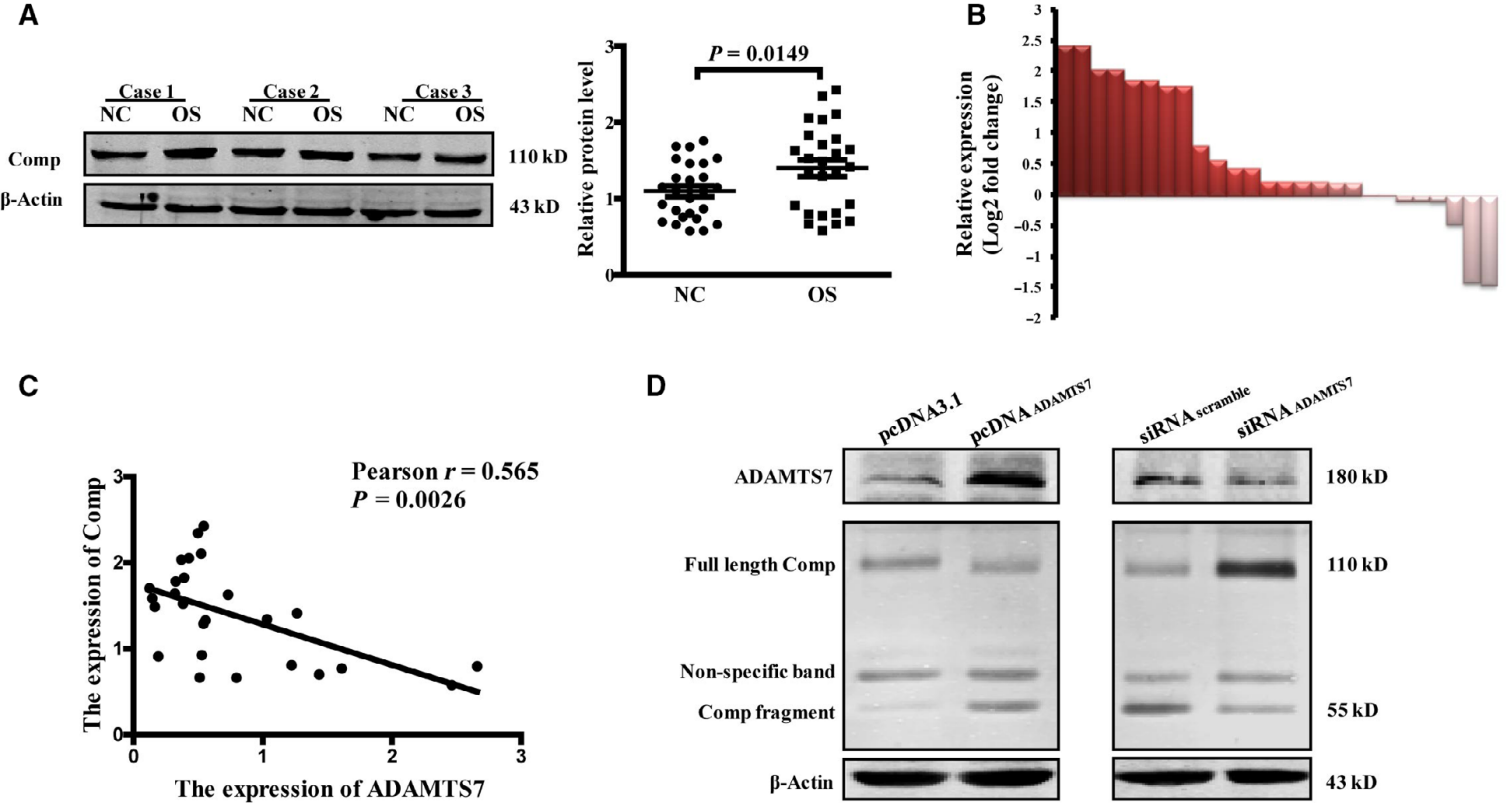
Comp was degraded by ADAMTS7 in OS cells. (A) Western blotting of Comp protein levels in OS tissues and adjacent tissues (*n* = 26). *P* value by Student's *t*‐test The relative expression level was quantified. (B) Protein expression levels are presented as the log_2 _(fold‐change)in OS samples relative to the adjacent tissues. (C) Correlation of ADAMTS7 and Comp protein in OS tissues. (D) Representative images of western blotting for full‐length Comp and degradation fragment in MG63 cells after ADAMTS7 overexpressing or silencing (*n* = 3).

Interestingly, we found Comp, a well‐known substrate of ADAMTS7, was significantly increased in OS tissues compared to the adjacent tissues (NC vs. OS: 1.094 ± 0.0725 vs. 1.398 ± 0.1088, *n* = 26, *P *= 0.0149) (Fig. 5A,B). Clinically, the expression of Comp was negatively regulated by ADAMTS7 (*P *= 0.0026) (Fig. 5C). Furthermore, the degradation of Comp in OS cells was confirmed by western blotting (Fig. 5D). As shown by the results, the full‐length Comp was decreased and degradation fragment was increased in ADAMTS7 overexpressing MG63 cells. In addition, the expression of Comp fragment obviously declined in ADAMTS7 silencing MG63 cells, which suggested a reduction of degradation.

### ADAMTS7 inhibited OS progression and promoted osteogenic differentiation by degrading Comp

**Fig. 6:**
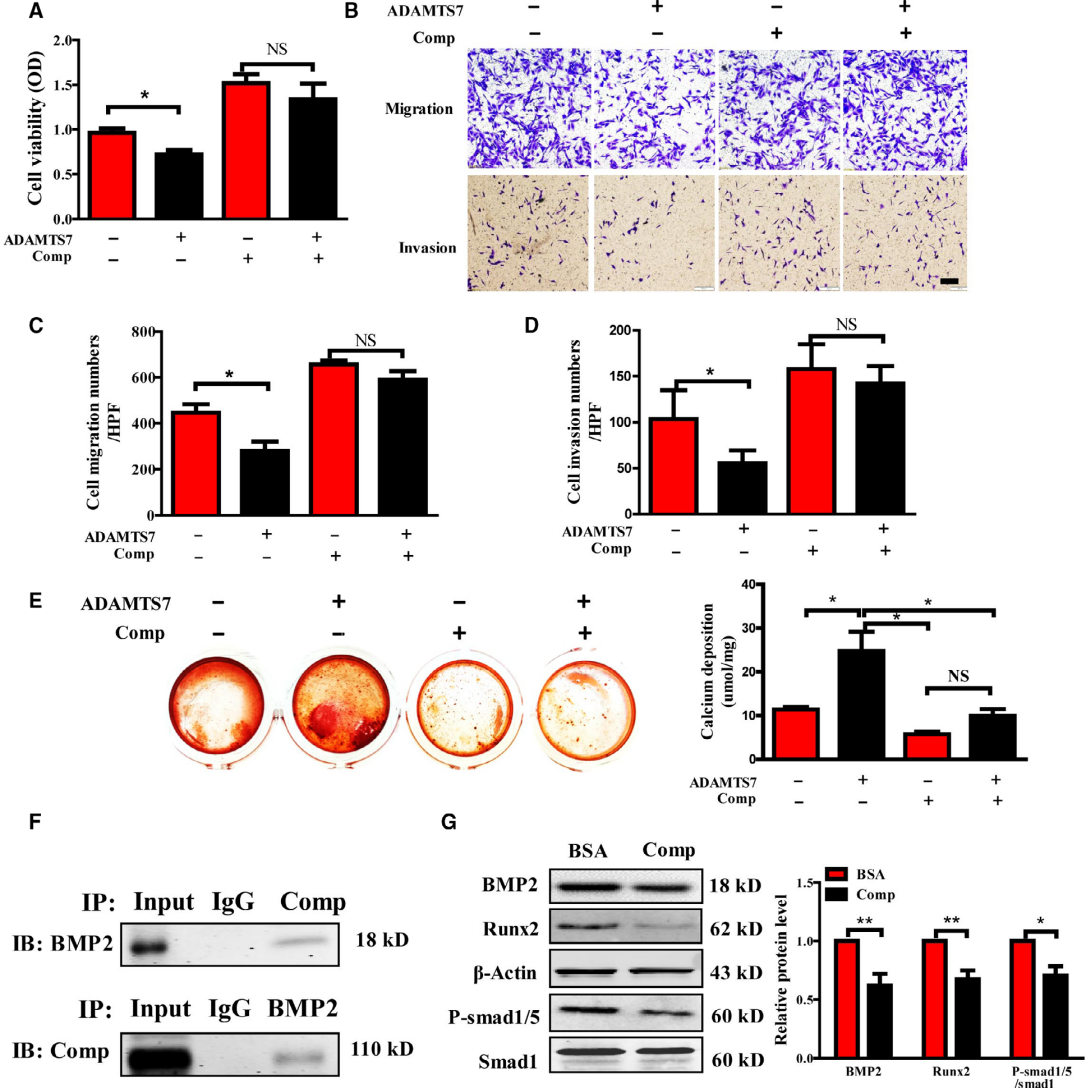
rComp mediated the effects of ADAMTS7 on OS cell proliferation, migration, invasion and osteogenic differentiation. (A) A CCK‐8 assay was conducted after 48 h to detect the proliferation of ADAMTS7 overexpressing MG63 cells with or without rComp (100 ng·mL^−1^). Values are expressed as the mean ± SEM from three independent experiments performed in duplicate. (B) A transwell assay was performed to detect the migration and invasion ability of ADAMTS7 overexpressing MG63 cells under rComp treatment (100 ng·mL^−1^). Scale bar = 200 μm. The number of migrated cells (C) and invaded cells (D) was calculated and quantified as the mean ± SEM for three independent experiments.*P < 0.05. NS, not significant. (E) Biomineralizations were measured in MG63 cells to evaluate the effect of rComp on the osteogenic differentiation triggered by ADAMTS7. Calcium deposition was analyzed and quantified as the mean ± SEM (*n* = 3). *P < 0.05. NS, not significant. Data in (A), (C), (D) and (E) were analyzed by one‐way ANOVA with the Student–Newman–Keuls test for multiple comparisons. (F) Representative images of co‐immunoprecipitation for Comp and BMP2. IB, immunoblot. (G) Representative images for western blotting of osteogenic markers in MG63 cells with or without rComp (100 ng·mL^−1^) pretreatment. The relative expression of BMP2, Runx2 and p‐Smad1/5 was quantified as the mean ± SEM for three independent experiments. **P < 0.01, *P < 0.05 by two‐way ANOVA with Bonferroni's test.

To determine whether the negative effects of ADAMTS7 on the malignant phenotypes of OS cells attributed to the interaction with Comp, proliferation and migration assays were further conducted in ADAMTS7 overexpressing MG63 cells with the stimulation of rComp. As shown in Fig. 6A, Comp significantly reversed the suppressed proliferation ability of ADAMTS7 overexpressing MG63 cells. The reduced migration and invasion ability could also be rescued by Comp (Fig. 6B,D). Moreover, Comp addition attenuated mineralized calcium phosphate nodule formation triggered by ADAMTS7 (Fig. 6E). Furthermore, the protein–protein interaction between Comp and BMP2 was confirmed by co‐immunoprecipitation in MG63 cells (Fig. 6F). As expected, rComp can reversely regulate the expression of osteogenic differentiation markers BMP2, Runx2 and p‐Smad1/5 in OS cells (Fig. 6G).

## Discussion

ADAMTS is a zinc‐dependent metalloproteinase family that plays a critical role in the assembly of the extracellular matrix and the shedding of membrane‐bound receptor molecules in a variety of physiology and pathology process [16, 22]. Although ADAMTS enzymes have been proposed to be crucial factors in cancer progression either in a positive or negative manner, recent findings and knowledge of ADAMTSs in OS progression is limited and shallow [23]. In the present study, we demonstrated for the first time that ADAMTS7 was down‐regulated in OS tissues. The low expression of ADAMTS7 was obviously associated with the adverse clinical outcomes, including poorer histological differentiation and a more advanced clinical stage, suggesting that ADAMTS7 may be involved in the pathogenesis of OS.

The reduced expression of ADAMTS7 in OS raised intriguing questions regarding its role in OS tumor biology. In the present study, we first demonstrated that ADAMTS7 refrained the oncogenic phenotypes of OS cells. Interestingly, the expression of ADAMTS7 in OS was positively associated with BMP2. BMP2 is a key regulator in bone tissue formation and remodeling [24]. Dysregulation of osteoblast differentiation is a key pathological process in OS. The relevance between ADAMTS7 and BMP2 gave rise to the possibility that ADAMTS7 functions as a potential inducer of differentiation in OS. We assessed the effects of ADAMTS7 on OS cell proliferation, migration and invasion through gain‐ or loss‐function studies in two OS cell lines, and revealed an innovative effect of ADAMTS7 with respect to inhibiting malignant phenotypes of OS cells. Furthermore, we found that ADAMTS7 silence repressed cell osteogenic differentiation, whereas the overexpression of ADAMTS7 could trigger OS cells transferring to the phenotype of osteoblasts, as illuminated by biomineralization and expression of osteogenic markers in OS cells. We also found the addition of Noggin could circumvent the inhibitory effect of ADAMTS7 on the malignant behavior of OS cells, suggesting ADAMTS7 might prevent OS progression via BMP2. Taken together, these findings demonstrate that ADAMTS7 served as a new anti‐oncogenic regulator in human OS, at least in part, by orchestrating osteogenic differentiation. Given that the use of primary cell lines from patients is better for addressing the issues of OS heterogeneity and pathological differentiation, we aim to further confirm the effects of ADAMTS7 by patient‐derived cell lines in the future.

Comp, also known as thrombospondin‐5, is a secreted matricellular protein that participates in a wide variety of physiologic or pathologic processes, such as chondrocyte proliferation, thrombin inhibition, collagen fibrillogenesis and the mechanical strength of tendons [25]. Mutations of *Comp* gene can cause two autosomal dominant chondrodysplasias: pseudoachondroplasia, which is a severe dwarfing condition and multiple epiphyseal dysplasia, which is a milder short stature disorder [26]. Comp also plays critical roles in maintaining cardiovascular homeostasis by maintaining vascular smooth muscle cell contractile phenotypes [27]. However, the effects of Comp on OS have never been revealed until now. In the present study, we found the expression of Comp increased in OS tissues and ADAMTS7 could degrade Comp in OS cells. Moreover, the addition of rComp could significantly abolish the osteogenic effect of ADAMTS7. Our results support the notion that Comp was responsible for ADAMTS7‐mediated OS suppression. As an endogenous BMP2 inhibitor, Comp can enhance osteogenesis by directly binding and activating BMP2 in the C_2_C_12_ cell line and in primary human bone mesenchymal stem cells, as well as in a rat ectopic bone formation model [28]. Overexpression of Comp inhibits BMP2‐induced osteogenic differentiation and promotes BMP2‐induced chondrogenic differentiation in mesenchymal stem cells [29]. Furthermore, Comp inhibits vascular smooth muscle cell calcification by interacting with BMP2 [30]. In the present study, we further revealed the interaction of Comp and BMP2 protein in OS cells and revealed that the inhibitory effects of ADAMTS7 on OS cell growth and metastasis relied on targeting Comp. However, further *in vivo* studies are needed to provide direct evidence of ADAMTS7/Comp/BMP2 axis in OS pathogenesis.

In summary, our findings provide new insight with respect to ADAMTS7 deficiency in OS cells inhibiting osteogenic differentiation by regulating the ratio of Comp and BMP2, ultimately accelerating cell growth and migration. These results suggest that ADAMTS7/Comp/BMP2 may be a novel therapeutic target for OS.

## Conflicts of interest

The authors declare that they have no conflicts of interest.

## Author contributions

CW and LZ conceived the study and acquired the funding. HX performed the formal analysis. YC and QT participated in the investigation. YC and XW contributed to the methodology. CW and LZ administered the project. BC and XM supervised the study. LZ wrote the original draft of the manuscript. XM wrote, reviewed and edited the manuscript.

## Data Availability

The raw data are available from the corresponding author upon reasonable request.
